# The impact of myocardial compressibility on organ-level simulations of the normal and infarcted heart

**DOI:** 10.1038/s41598-021-92810-y

**Published:** 2021-06-29

**Authors:** Hao Liu, João S. Soares, John Walmsley, David S. Li, Samarth Raut, Reza Avazmohammadi, Paul Iaizzo, Mark Palmer, Joseph H. Gorman, Robert C. Gorman, Michael S. Sacks

**Affiliations:** 1 James T. Willerson Center for Cardiovascular Modeling and Simulation, The University of Texas at Austin, Austin, TX USA; 2 Engineered Tissue Multiscale Mechanics and Modeling Laboratory, Virginia Commonwealth University, Richmond, VA USA; 3 Computational Cardiovascular Bioengineering Lab, Texas A&M University, College Station, TX USA; 4 Visible Heart Lab, University of Minnesota Twin Cities, Minneapolis, MN USA; 5 Gorman Cardiovascular Research Group, University of Pennsylvania, Philadelphia, PA USA; 6 Corporate Core Technologies, Medtronic, Inc., Minneapolis, USA

**Keywords:** Biomedical engineering, Cardiology

## Abstract

Myocardial infarction (MI) rapidly impairs cardiac contractile function and instigates maladaptive remodeling leading to heart failure. Patient-specific models are a maturing technology for developing and determining therapeutic modalities for MI that require accurate descriptions of myocardial mechanics. While substantial tissue volume reductions of 15–20% during systole have been reported, myocardium is commonly modeled as incompressible. We developed a myocardial model to simulate experimentally-observed systolic volume reductions in an ovine model of MI. Sheep-specific simulations of the cardiac cycle were performed using both incompressible and compressible tissue material models, and with synchronous or measurement-guided contraction. The compressible tissue model with measurement-guided contraction gave best agreement with experimentally measured reductions in tissue volume at peak systole, ventricular kinematics, and wall thickness changes. The incompressible model predicted myofiber peak contractile stresses approximately double the compressible model (182.8 kPa, 107.4 kPa respectively). Compensatory changes in remaining normal myocardium with MI present required less increase of contractile stress in the compressible model than the incompressible model (32.1%, 53.5%, respectively). The compressible model therefore provided more accurate representation of ventricular kinematics and potentially more realistic computed active contraction levels in the simulated infarcted heart. Our findings suggest that myocardial compressibility should be incorporated into future cardiac models for improved accuracy.

## Introduction

Myocardial infarction (MI) causes irreversible damage to cardiac muscle due to cessation of local blood flow following coronary artery occlusion. Approximately 1.5 million people suffer MI annually in the United States, with more than 15.9 million cases occurring globally. MI impairs cardiac contractile function within 1 min, and the subsequent post-ischemic event cascade is triggered almost immediately without rapid intervention^1^. MI induces myocardial fibrosis, which includes significant compositional, structural, and functional changes during the transition from infarcted myocardium to scar tissue^2^. This maladaptive remodeling process is typically severe and irreversible, leading to dilatation of the left ventricle (LV), wall thinning, papillary muscle displacement, mitral valve leaflet tethering and regurgitation, and mitral annular dilatation^3^. These profound pathophysiological changes collectively induce progressive heart failure (HF)^4^. Current treatment strategies for MI-induced remodeling and HF continue to rely primarily on empirical clinical experience and there is an urgent need for novel therapeutic modalities, studies of treatment duration and dosing, and design of mechanical supports that can be personalized for each patient.

In silico modeling is becoming a major tool for improving our fundamental understanding of HF and developing patient specific therapies^5^, and great progress has been made in organ-level cardiac modeling to describe the diverse aspects of heart biomechanical function, electrophysiology, heart disease, and treatment^6–8^. Current application areas include the etiology and pathophysiology of myocardium remodeling, its impact on tissue-level properties and organ-level cardiac function, and improvement of virtual surgery technologies and medical device development. In silico modeling can also provide quantitative risk stratification tools for these interventions^9^. A patient-specific computational platforms that can accurately predict the effects of MI on cardiac function, both acutely and chronically, would therefore be extremely valuable for developing and determining treatment strategies. However, simulation approaches must rest on a firm understanding of the function of myocardial tissue function in health and disease, which remains an active area of research^10^.

Given the thick-walled structure of the heart, a fully three-dimensional approach to modeling myocardium mechanical behavior is clearly necessary. Recent studies have explored the passive state in cuboidal specimens^11–15^. In particular, through the use of full 3D kinematics, triaxial structural-mechanical measurements, and finite element modeling, complex 3D coupling behavior between myofibers and collagen fibers in the extracellular matrix has been revealed. In contrast, only a single multi-axial study has been performed on active contraction^16^. Specific passive and active mechanics were found to be different, i.e., passive properties are more compliant and markedly nonlinear, whereas the activated properties are substantially stiffer and more linear. Clearly, the development of computational models of myocardial behaviour remains a challenging area, as several fundamental phenomena remain incompletely understood.

One such area is the interrelationship between myocardial deformation and perfusion pressure. Studies of ex vivo perfusion pressure in isolated and arrested hearts have demonstrated that: (i) myocardial deformation is not isochoric (i.e. not volume-preserving), and perfusion pressurization produces substantial radial thickening and increase in myocardial tissue volume (up to 15%, greatest at the endocardium) associated with an increase in intramyocardial capacitance; and (ii) there is decrease of myocardial compliance (or stiffening) in the longitudinal and radial directions^17^. Thus, volumetric changes are strongly correlated with the anisotropic myofiber fiber architecture and the microvessels aligned with these fibers rather than the larger transverse transmural vessels. If increases in perfusion pressures modulate myocardial volume, then intuitively, actively contracting myocardium should also be able to drive blood in and out of the coronary vasculature. Despite their functional importance, the exact effects of contractile forces on blood flow in the coronary vessels, microcirculation, and venous collection systems over the cardiac cycle remain unclear, as does their relation to myocardial volume changes during systole. The passive response of myocardium is commonly modeled either as a single-phase, nonlinear anisotropic incompressible hyperelastic solid, or one with embedded cylindrical vessels of known distensibility and varying diameter as function of the perfusion pressure^18^. The well-established “garden hose” effect, i.e., altered coronary perfusion changes the apparent stiffness of the tissue, is described by this later class of models. More formal modeling frameworks employ the theory of poroelasticity, which treats the myocardium as a mixture, with the goal of capturing the interactions between the solid and fluid phases. For myocardium, poroelasticity has been employed to describe observed viscoelastic behavior due to fluid filtration in the extracellular space and blood vessels^19^, to employ muscle contraction as the driving force for blood flow^20^, and to describe stiffening with increase in fluid pressure^21^.

**Figure 1:**
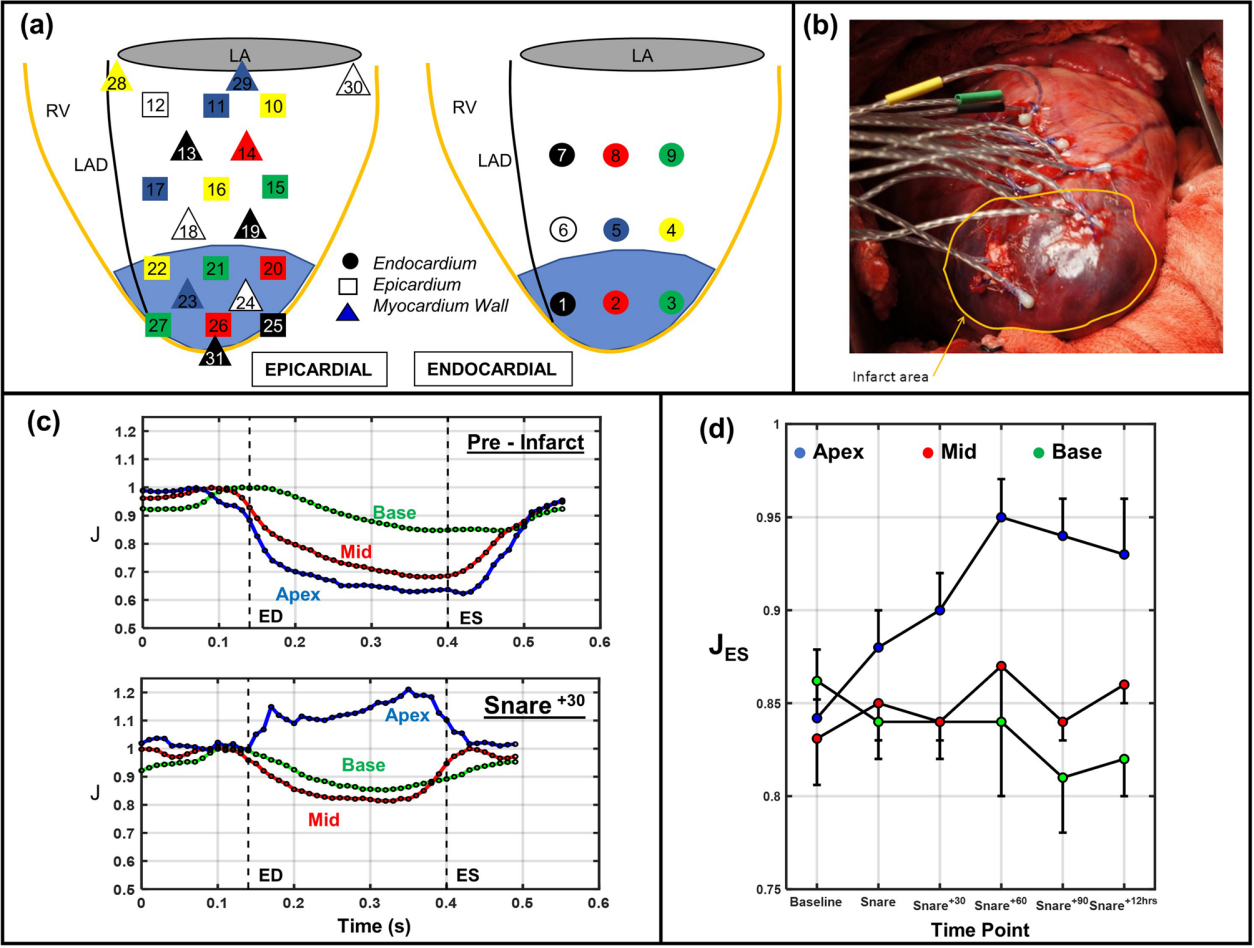
(**a**) Schematic showing sonocrystal array on endocardial and epicardial surfaces, (**b**) in-vivo sonocrystal deployment, (**c**) temporal changes of myocardial volume for apical, middle, and basal regions in one cardiac cycle: (top) pre-infarct, (bottom) 30 min post-infarct, (**d**) time evolution of volumetric change for apical, middle, and basal regions in both pre-MI and post-MI state.

To provide additional insight into the nature of local myocardial compressibility, we recently conducted a novel experimental study to quantify local ($\sim$1 cubic centimeter, cc) 3D time-evolving myocardium volume changes over the cardiac cycle (Fig. 1a, b)^22^. Results demonstrated a progressive reduction in myocardial volume from end-diastole (ED) to end-systole (ES), with a normalized volume $\sim$0.80 at ES. Regionally, volume reductions increased from base to apex (Fig. 1c). We also investigated how the time course behavior of MI impacted local contraction and found that myocardium tissue volume reduction was substantially affected (Fig. 1d). Specifically, ischemic impairment of contractility attenuated myocardial volume reduction acutely in systole, and was restored upon subsequent reperfusion. However, the recovery subsided as early as 60 min after reperfusion (Fig. 1d). Taken as a whole, these and previous results clearly indicate that myocardium does not behave isochorically over the cardiac cycle at the organ level. In addition to its role in wall kinematics, we hypothesize that myocardial compressibility can have a significant influence on simulated cardiac energetics (myofiber force generation levels) and contractile function (ventricular kinematics).

The objective of this study was thus to investigate the impact of incorporating a more physiologically accurate myocardial material model that includes these effects of compressibility for improving the accuracy of organ-level cardiac simulations. We first developed a high-fidelity in silico biventricular cardiac model using an extensive dataset collected from a single ovine heart to minimize issues caused by heterogenous data sources. A specialized compressible myocardial tissue constitutive model was then developed to allow for strictly controlled volume reductions due to active contraction during systole. This cardiac model was used to simulate whole-cycle cardiac kinematics in the normal and infarcted heart, including the effects of synchronous versus temporally- and spatially-varying electrophysiologically-driven contraction. Results of the simulated kinematics are then validated using sonocrystal-derived strains and in vivo echocardiographic imaging, and used to assess the impact of compressibility on myocardial kinetics.

## Results

### A single-heart ovine cardiac model

**Figure 2:**
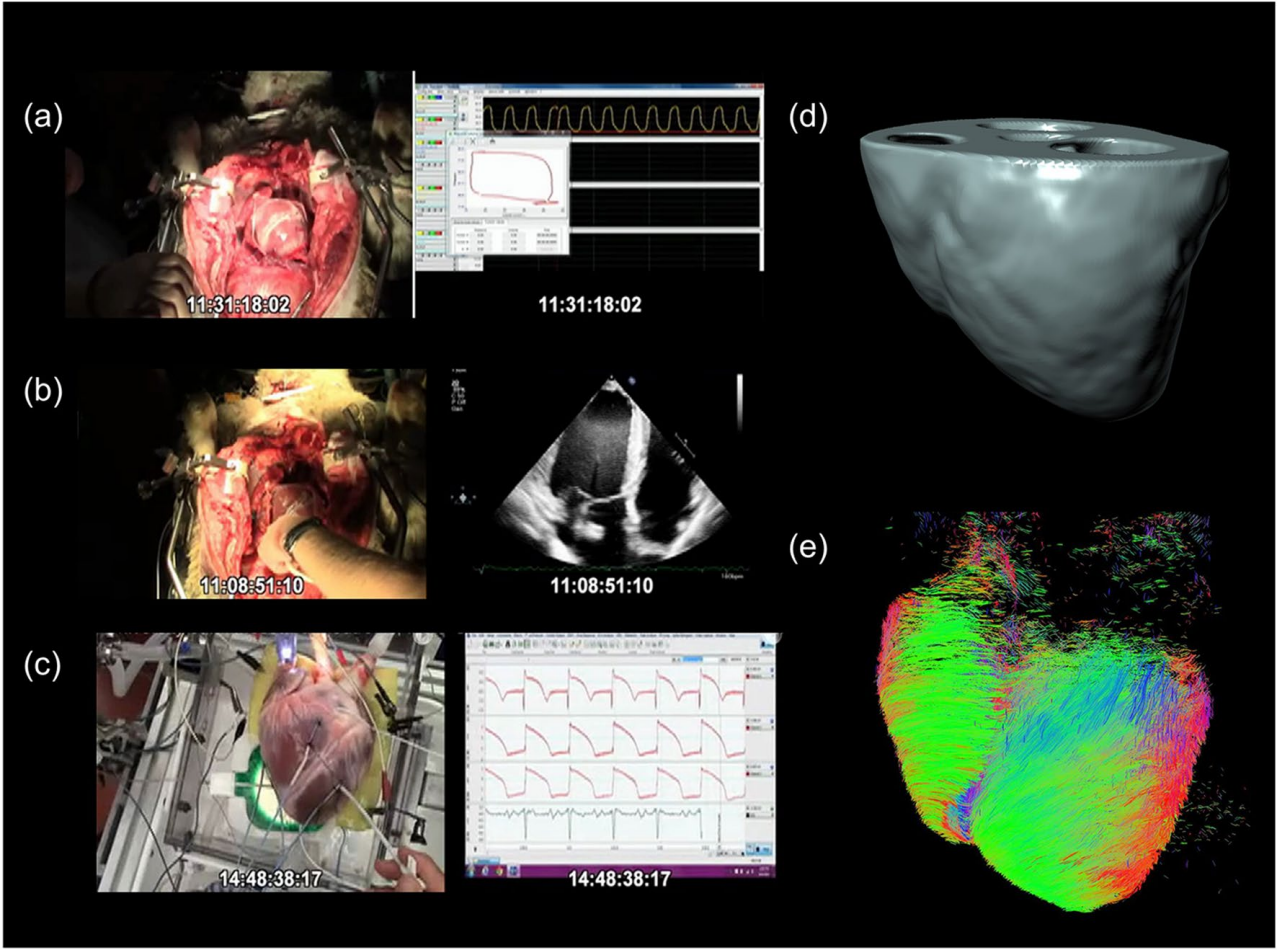
Data collection from a single heart for in silico model development. (**a**) Pressure volume loops, (**b**) echocardiography, and (**c**) epicardial monophasic action potentials were obtained in vivo and subsequently ex situ. (**d**) MRI and (**e**) DTMRI were employed to obtain accurate descriptions of anatomy (for FE mesh construction) and myofiber directions (to assign material heterogeneity and anisotropy).

**Figure 3:**
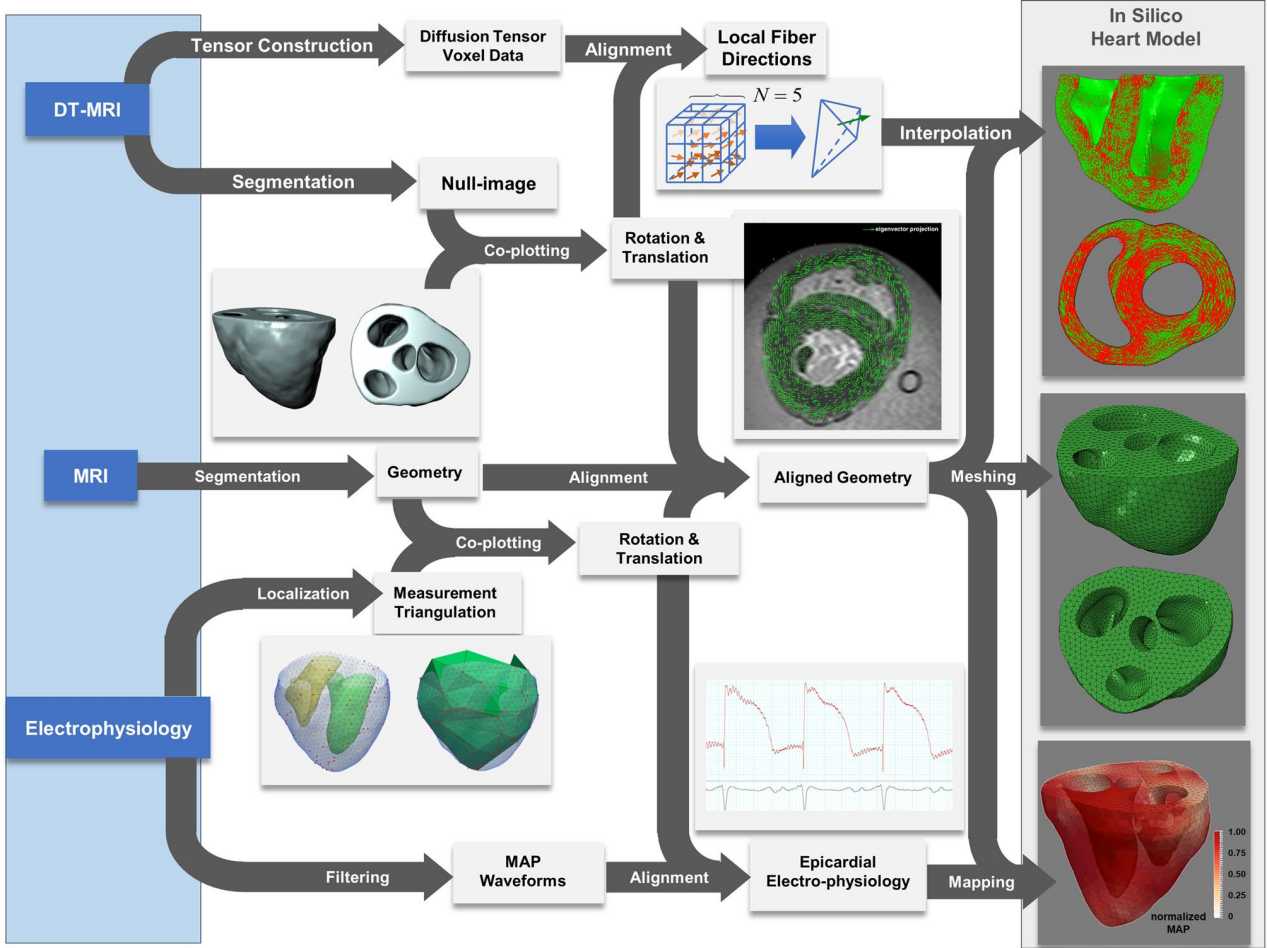
Extensive datasets collected from a single ovine heart (left light blue panel) were employed in a pipeline to develop the in silico heart model. MRI, DTMRI, and temporal evolutions of MAPs were co-localized in a 4D FE mesh.

We developed an in silico model of cardiac function with and without MI based on extensive datasets from a single ovine heart collected in vivo, ex situ, and ex vivo (Figs. 2 and 3), including image and data registration, regional heterogeneity, electrophysiology, and organ-level function integration (see Methods). The employment of a complete dataset from a single heart avoided the need to match datasets from different animals and/or prescribed models. Briefly, heart cavity pressures and volumes were acquired in vivo with catheterization and sonomicrometry (Fig. 2a). Diverse echocardiographic imaging was conducted to obtain validation datasets of cardiac function in vivo (Fig. 2b). Epicardial monophasic action potentials (MAPs) were measured using a grid of contact electrodes (Fig. 2c). The ex situ step was conducted in an isolated heart flow loop, replicating in vivo conditions and enabling extensive data collection (Fig. 2c). Subsequently, the heart was pressurized, fixed and gelled at end-diastolic pressure for imaging. Magnetic resonance imaging (MRI) scans were then segmented to create a finite element (FE) mesh (Fig. 2d), and diffusion tensor MRI (DTMRI) data was employed to prescribe principal myofiber directions for the specification of anisotropic mechanical properties and active contraction (Fig. 2e).

### A uni-directional compressible myocardial material model

The mechanical behavior of passive myocardium was described using a transversely isotropic Fung-based hyperelastic model for the deviatoric component, with four parameters. Parameter values were then fitted to the Klotz end-diastolic pressure–volume curve^23,24^, and the volumetric response was described by the bulk modulus. Active mechanics were modeled using an additive contractile stress along the myofibers^25^. We modeled the distinctive compressible material behavior observed in the myocardium by assuming it to be nearly incompressible in diastole (thus preventing further volume increases), but compressible in systole to allow for volume reductions while actively contracting^22^. Compressibility was incorporated through a specialized material model that utilized a penalty term allowing for only reductions in volume modulated by active contraction. That is, the bulk modulus was not a material constant, but instead, a material function that decreased with the current amount of local active fiber stress.

### Establishing the end-diastolic pressure volume relationship

In the passive (diastolic filling) state, myocardium can be considered functionally incompressible. Thus, passive material parameters were calibrated such that the organ-level model reproduced a general end-diastolic pressure–volume relationship (EDPVR)^24^. Because the end-diastolic configuration employed to generate the heart geometry was pressurized (at $\sim$15 mmHg), this configuration could not be considered as the stress-free reference configuration. Thus, we employed an inverse FE approach to obtain a reference configuration that, when subjected to end-diastolic pressure, would match the imaged configuration. This procedure was necessary because traditional FE analyses require stress-free reference configurations, and the computed deformations refer to the state at which the true ventricular filling pressure is zero with no transmural pressure gradients^26^. The passive model was matched to the general EDPVR, with the fitted constants in agreement with previous results^23,27–30^.

### Active mechanics of the pressure–volume loop

The temporal and spatial heterogeneity of calcium transients estimated from epicardial MAPs drives active contraction in the asynchronous simulations (see Methods and MOVIE01 supplementary material). These local patterns of force generation *f*(*x*, *t*) are then multiplied by the global stress due to contraction $T_{\mathrm{Ca}^{2+}}(t)$ and local strain. In the synchronous case, $f(x,t) = 1$ everywhere. The problem then reduces to fitting the active force $T_{\mathrm{Ca}^{2+}}(t)$ in the myocardium necessary to generate the experimentally observed left ventricular volume at time *t* when the measured pressure at time *t* is applied to the endocardial surface.

**Figure 4:**
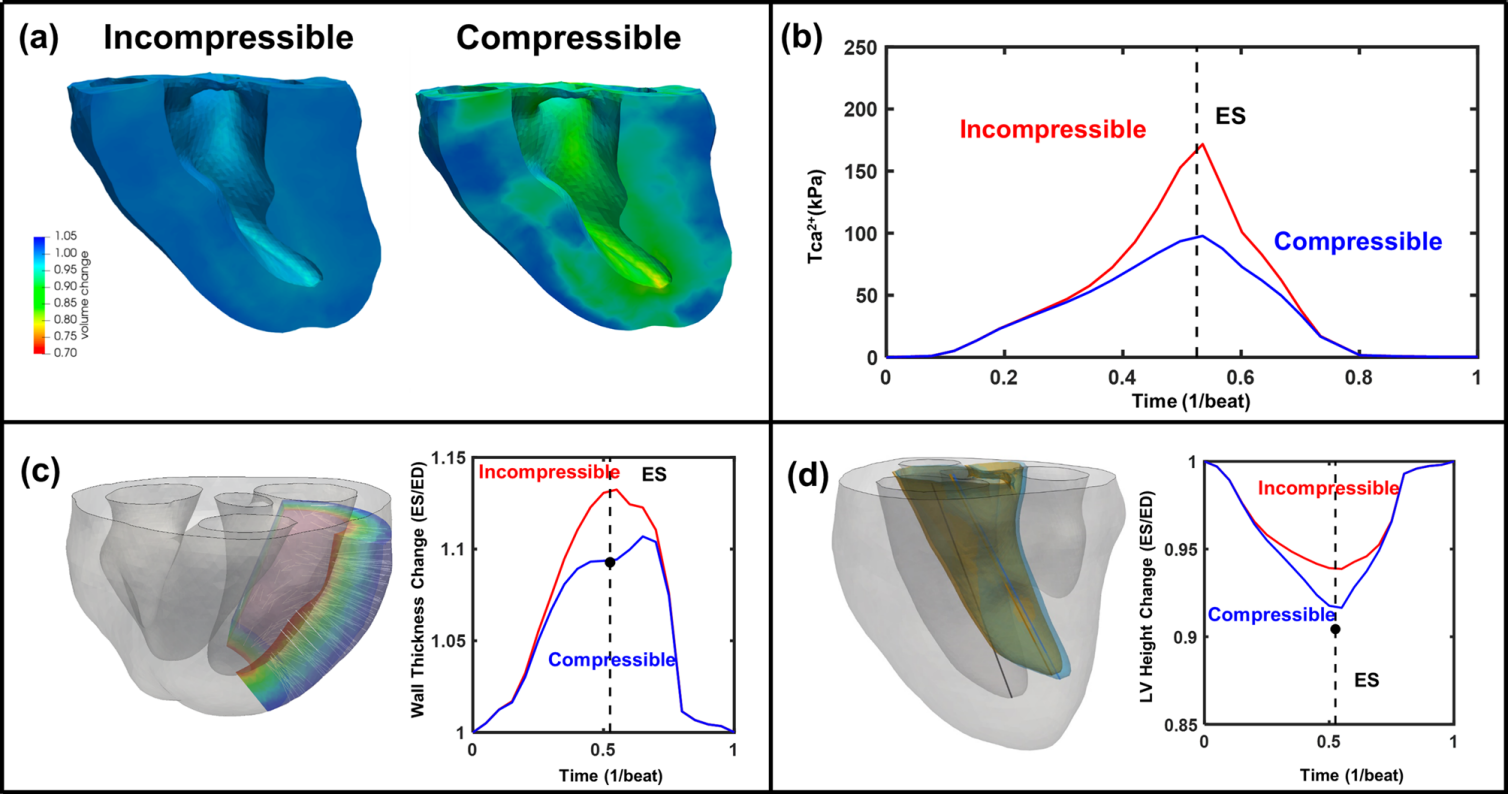
(**a**) Comparisons of predictions in volume change (end-systole) between the compressible and incompressible models, (**b**) $T_{\mathrm{Ca}^{2+}}$ fitted by the compressible and incompressible models, (**c**) LV free wall (LVFW) thickness change distribution for compressible and incompressible models, and black dot in end-systole means validation point from sonocrystal study, (**d**) LV height measured from the compressible and incompressible models. The black dot at end-systole represents the validation data point from in-vivo 2D echocardiography.

### Active contraction and the estimation of active myofiber stress

The incompressibility assumption enforces isochoric deformation of the myocardium, whereas the compressible model experienced transmural changes in myocardial volume (Fig. 4a). The incompressible model necessitated a much larger contraction force to replicate the measured PV loop, resulting a two-fold increase in the peak active stress during systole (172 kPa in the incompressible model vs. 98 kPa in the compressible model) (Fig. 4b). This finding arises primarily from lateral bulging of the incompressible material when contracting/shortening along the fiber direction. In contrast, a compressible material can undergo considerable shortening without expansion in the cross-fiber direction. The compressible myocardium in silico heart model allowed for the reduction of volume due to active contraction (down to 80% of the original volume, with a transmural average of around 10% volume reduction). The compressible model provided closer predictions of global wall thickening and change in LV length from ED to ES as compared to our validation data (Fig. 4c, d), while the incompressible model overestimated wall thickening and underestimated LV shortening at ES.

The incorporation of asynchronous active contraction patterns impacted both active stresses and volumetric change in normal myocardium. Introducing asynchronous contraction caused heterogeneity in the regional active stress estimated in the myocardium, whereas the active stress in the synchronous case was very uniform [Fig. 6 (top panel)]. Minor variations in active stress in the sychronous case arose due to regional differences in the myocyte stretch $\lambda$. The estimated myofiber active stress could be up to 1.5 times in asynchronous contraction when compared with models with synchronous contraction. Furthermore, asynchronous contraction caused more pronounced variation in myocardial volume changes throughout systole as compared to the synchronous case, and this effect was most prominent at the endocardium [Supplemental Figure (top panel)]. In general, myocardial compression was increased with asynchronous contraction, although this effect was very spatially heterogeneous. Despite regional variations in stress, the global average active stress was very similar between the synchronous and asynchronous compressible simulations. In particular, the difference in the global active stress between the two compressible simulations was an order of magnitude smaller than the difference with the incompressible simulations (Fig. 7).

**Figure 5:**
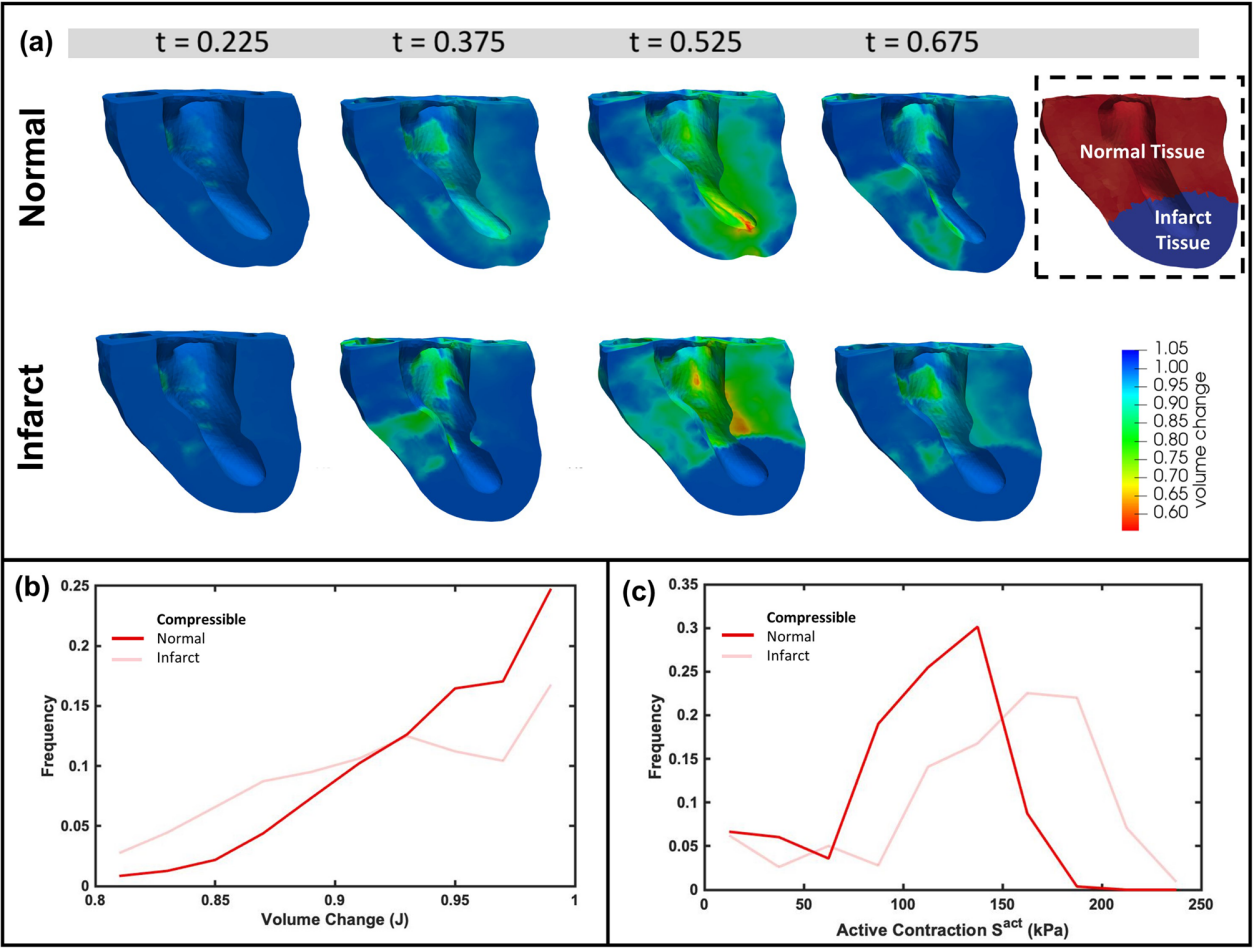
(**a**) Comparisons of volume reduction in one cardiac cycle between the normal and infarcted heart models, (**b**) distribution of spatial volume change within normal tissue from the normal and infarcted models, (**c**) distribution of spatial active contraction within normal tissue from the normal and infarcted models. All times *t* are normalized to cycle duration.

### Effects of myocardial infarction

**Figure 6:**
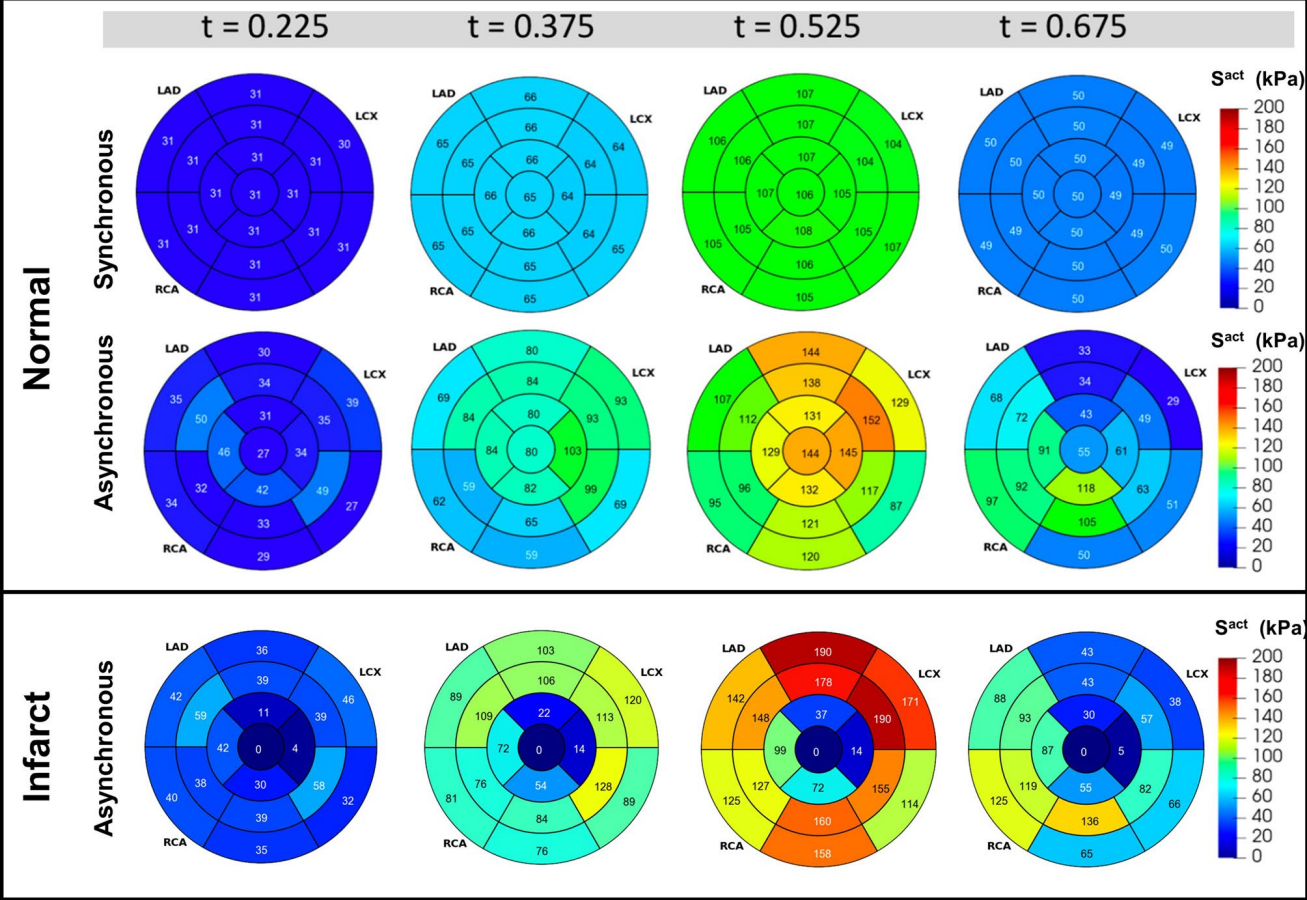
Effect of myocardial infarction and synchronous vs asynchronous on active stress distribution over the course of one cardiac cycle. Endocardial data are shown using a standard AHA segmentation of the LV. All times *t* are normalized to cycle duration.

**Figure 7:**
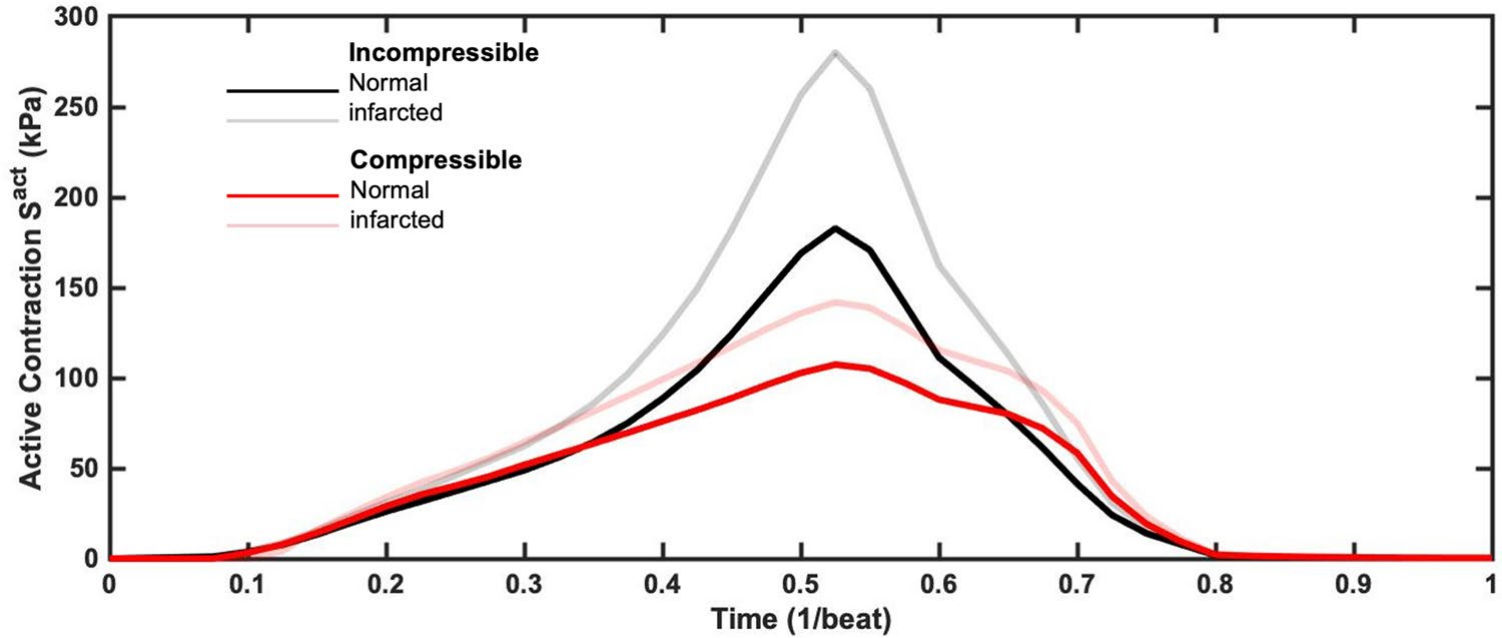
Effect of myocardial infarction and compressibility on active stress distribution within one cardiac cycle. Here, accounting for tissue compressiblity resulted in a pronounced reduction in estimated active myofiber stress in both the pre- and post-MI states.

In the final set of simulations, we investigated the effects of MI as performed in the sheep dataset by setting the active stress $S^{act} = 0$ in Eq. (8) within the infarcted myocardium. We simulated an apical infarct as in the experiment, where pump function was impaired by MI. We observed substantially different LV kinematics when MI was simulated, with compensatory mechanisms enabling more vigorous contraction of the remaining normal myocardium, since the infarcted myocardium deforms purely passively (Fig. 5a, b). The redistribution of active stress is shown in histogram plot (Fig. 5c) and in the AHA segmentation bullseye plots in Fig. 6 (bottom panel). Note that the AHA segmentation does not align exactly with the infarct location, giving rise to a small active stress in some apical segments due to normal myocardium also being present. The compressible model required a relatively small adjustment in contraction force in the remaining healthy myocardium to compensate for loss of contractility in the infarcted region and maintain pump function [107.4 kPa peak systolic active stress in the normal heart versus 141.9 kPa in the MI case, (Fig. 7a)]. In contrast, the incompressible model with MI required a much greater relative increase (53.3%) (Fig. 7c) and absolute increase (97.4 kPa) (Fig. 7b) in peak systolic active stress to generate the experimentally recorded PV loop.

### Experimental validation

To validate the simulated effects of compressibility on the resultant simulated cardiac kinematics, sonomicrometry data from our previous study was utilized^22^ to compute wall thickness changes in the LV free wall between ED and ES (see Methods for details). Wall thickness derived from the simulations for incompressible case was approximately 1.133, whereas the compressible case was 1.094, a value very close to the experimentally measured case of 1.0933 (Table 1).

The sonomicrometry measurements, the absolute difference and ratio between ED and ES state were in the middle between output from the incompressible and compressible models (Table 1). The assumption of incompressibility results in almost double the absolute change in wall thickness during systole as compared to the compressible model and experimental data. Incompressibility also gives a 5% higher ratio of wall thickness from ED to ES. As a further validation step, we also determined LV length from echocardiographic data recorded in vivo from the sheep used to construct the computational model (Table 1). We found that the compressible material model gave a much closer match to this data than the incompressible material model, as the compressible material model produced a more pronounced systolic shortening in line with the experimental data.

**Table 1:** Comparisons of wall thickness change of LV free wall and shortening of LV height between in-silico models and validation data.

Parameters	Groups	Subgroups	ES/ED
LVFW thickness	In-silico models	Incompressible	1.133 ± 0.059
		Compressible	1.094 ± 0.043
	Validation	Sonoxtals	1.093 ± 0.0321
LV height	In-silico models	Incompressible	0.939
		Compressible	0.917
	Validation	2D Echo	0.904

## Discussion

The primary goal of the present study was to evaluate the effects of myocardial compressibility on simulated heart function. A detailed in silico model built from a single-ovine-heart was developed, with results validated using both in vivo imaging and sonocrystal data. Compared to an incompressible myocardial material model, the compressible material model demonstrated improved agreement in both ventricular geometry and myocardial tissue kinematics (LV free wall (LVFW) thickness and LV height), and a $\sim$50% reduction in peak active stress generation during contraction. Importantly, when the immediate post-MI state was simulated, the change in the average active tension global was much smaller than that predicted with the incompressibility assumption. We also examined the effects of synchronous and asynchronous excitation-contraction patterns derived from epicardial MAP recordings. The cardiac model with synchronous contraction was inherently unable to depict accurate functional behavior, and the approximated asynchronous contraction improved the agreement with measured LV function.

In the current study, we sought to model the myocardium strictly at the organ level, considering a local volume scale of $\sim 1 cc$^22^. The resulting accurate accounting of myocardial compressiblity at the $\sim 1 cc$ scale lead to clear improvement in predicted wall thickness and LV height (See Fig. 4 and Table 1). For example, wall thickness change estimates improved from the incompressible model value of 1.133 to the compressible model value of 1.094 (experimental value was 1.093). While an absolute difference of $\sim 5\%$, it represents a proportional change of about 42% with respect to the actual measured value. These kinematic results are not at all surprising when considering that incompressible and compressible materials responses are fundamentally different. Contracting incompressible materials undergo volume preserving deformations and have to laterally “bulge out” to compensate for the contractile strain along the myofiber directions (e.g. a unit cube actively contracting to 80% of its original length thickens to 112% its original width). In contrast, contracting compressible materials undergo volume reductions when compressed and are able undergo similar active contractions with lateral thickening of only 102%. This result may be relevant to cardiac mechanics in terms of the relationship between fiber shortening and cross-fiber thickening. This relationship is crucial for the estimation of contractile forces since less energy is necessary if fewer cross-directional interactions occur between myocytes.

Next, we examined these effects of affected local myocardial volume at end-systole ($J_{ES}$) in the immediate post-MI period. As shown in Fig. 1, $J_{ES}$ in the infarct region (i.e. the apex region in this animal model) *increased* from 0.84 in the pre-MI state to 0.95 at 60 min post-snare. This clearly demonstrated the effects of reduced perfusion on the local myocardial volume during systole. In addition, $J_{ES}$ in the base region (which is normal myocardium) *decreased* from 0.86 in the pre-MI state to about 0.825 in the same 90 min post-MI period. These experimentally measured features were both significant and experimentally verified in the computational results (see Figs. 4a and 5a) using the compressible material model. Moreover, by linking systolic compressiblity to the degree of contraction, we were able to closely match the experimental observations. Our results are of particular relevance to estimation of active stress in organ-scale cardiac models derived from clinical cardiac imaging sources, which typically merge the vasculature with the myocardial tissue due to image resolution constraints.

**Figure 8:**
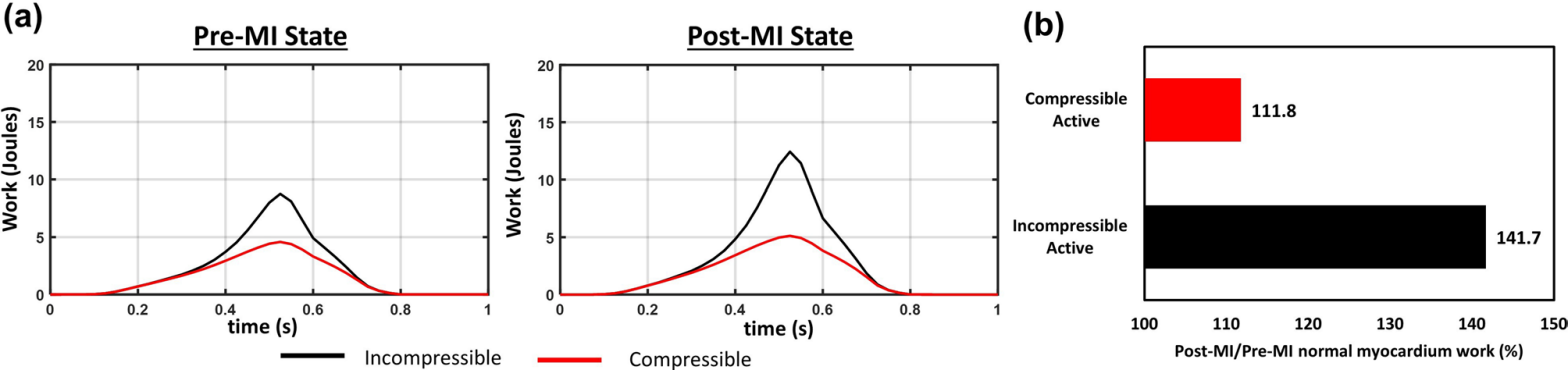
(**a**) Comparisons of work-time relation for normal myocardium between pre-MI state and post-MI state in compressible and incompressible models(black line: incompressible; red line: compressible). (**b**) The post-MI/pre-MI peak work ratio (expressed as a percent) for the incompressible and compressible model for the active component.

In addition to improving the accuracy of ventricular kinematics, use of a compressible material model had a substantial impact on the active fiber stress estimates pre- and post-MI (Fig. 7). This finding should be considered in context to the choice of scale on the computed 3D stress tensor. Using the $\sim 1 cc$ scale, the resulting stress tensor is a *homogenized average* across the major myocardial structures, including myofibers, connective tissue, and the vascular bed. We thus refer to computed active myofiber stress determined and interpreted in this manner only. This approach is consistent with that taken by others using incompressible material models, wherein the active contraction $\mathbf {S}^{act}$ levels were comparable^31–35^. The impact of the material model compressibility on organ-level computational modeling of the heart is more than just an improved *relative* of the estimated active fiber stress levels. To further demonstrate this, we computed the active work (in Joules) during systole for both material models (Fig. 8). Differences in the active work-time between the models was quite evident. Specifically, the incompressible model peaked at 8.770 J in the pre-MI state followed by 12.430 J in the post-MI state. In contrast, the compressible model peaked at only 4.583 J in the pre-MI state followed by 5.125 J in the post-MI state (Fig. 8a). The effects are made clearer when looking at the post-MI/pre-MI peak work level ratios, where the incompressible model predicted a 141.7% change whereas the compressible model only 111.8% change (Fig. 8b). We note that these changes were simulated with the same elastic material model and parameter values (excluding differences in the prescribed bulk modulus as a function of time), with the same geometry, boundary conditions, and fiber structures, and therefore represent the isolated effects of compressiblity. These results clearly demonstrated that compressibility has a substantial effect on active contraction behaviors in response to immediate post-MI state.

When considering the underlying mechanisms of myocardial volume change during systole, we note that volumetric changes in the myocardium during the cardiac cycle have been known to occur for over a century^36^. The length scale considered is also critical; use of smaller volumes of myocardium tend to contain a larger proportion of the smaller capillary network as opposed to larger vessels, which can lead to more modest volume reductions from ED to ES. In contrast, the larger volumes of myocardium considered herein are likely to contain principal branches of coronary arteries, leading to greater changes in volume during the cardiac cycle. Irrespective of the actual mechanism, we found changes in $J_{es}$ to be consistent across multiple animals (Fig. 1). Yet, the incorporation of this class of effects into organ level cardiac simulations and determining how they affect cardiac function remains under-investigated. The rationale for assuming incompressibility of myocardial tissues is that they are predominantly composed of incompressible solid constituents with a compartmentalized, effectively incompressible fluid phase. Arteries also undergo volume preserving deformations, an assumption is indeed justified^37^. Within the heart, the high energetic demands of the myocardium require an extensive coronary vasculature, with a total vascular tissue volume fraction of about 15–20%^38^. It has been shown using a viable ex vivo preparation that changes in coronary perfusion pressure induced near isotropic-like changes in LV wall deformations as the coronary volume was changed^39^. Pressure in the coronary artery tree and the turgor pressure of the myocardial microvasculature, modulated either by increasing the coronary venous resistance or coronary artery pressure, reversibly stiffens the LV^40^. McCulloch et al. noted that coronary perfusion cannot be neglected in an accurate continuum mechanics model of the passive left ventricle^41^. Perfusion alone may also affect the regional shape and tissue volume of the myocardium because a close structural coupling between the coronary microvasculature and the surrounding myocytes is thought to exist^17^.

Thus, blood perfusion and myocardium mechanics are deeply interconnected, yet the exact mechanisms responsible for the observed reductions in tissue systolic volume remain unclear. It is known that blood flow to the heart during systole is negligible^42^. Specifically, myocardial contraction compresses the vascular beds, which acts as a throttling mechanism that drives blood downstream into the venous drainage and coronary sinus, impeding further coronary flow perfusion^43^. This intricate interplay between myocardial contraction and the cardiac vasculature is also highly efficient; arteries and arterioles are often accompanied by two larger veins and venules forming a “triad”^44^. This arrangement promotes advantageous countercurrent exchanges of heat, gases, and nutrients^45^ and “protects” the arterial system, as preferential compression of the venules attenuates compression of the arterioles during systole and leads to more efficient perfusion^46^. Myocardial volume changes during the cardiac cycle may be functional, since they result in alternating systolic discharge and diastolic suction on arterial blood, i.e., an “intramyocardial pump”^47^, which has also been interpreted as time-varying elastance of the cardiac tissue^48,49^. In addition, remarkable differences in the effect of cardiac contraction on intramyocardial vessels exist between the sub-endocardium and the sub-epicardium, the former being subjected to greater increases in resistance as well as to larger displacements of blood^50,51^.

Despite the above evidence for some form of myocardial systolic compressibility, compressible models for myocardium are rare. Recently, compressibility and anisotropy of *excised, unperfused* myocardial tissue was investigated, which concluded that myocardial tissue is slightly compressible^52^, which supports the explanation of changes in vascular volume are responsible for the observed changes in $J_{es}$. While changes in vascular volume appear to be the most logical explanation, many questions remain. For example, is the systolic contraction-induced volume reduction mainly due to the venous return, or the arterial supply, or both? A poroelastic modeling approach may be appropriate here^53^, but best approaches to accurately account for the effects of systolic contraction remain unclear. We thus emphasize that in the present study we did not explicitly model the *underlying mechanisms* for the observed compressiblity responses. Recall too that at the organ level, stress is an homogenized average across the major myocardial structures, including myofibers, connective tissue, and the vascular bed. So, we only refer to active myofiber stress determined and interpreted in this manner. We thus restricted ourselves to model the *consequences* of the observed systolic compressiblity on organ-level kinematics and estimates of homogenized active myofiber stress patterns pre- and post-MI.

Our findings also demonstrate that further development of material models of the myocardium in cardiac simulations is still needed. Most constitutive models of myocardium are anisotropic, consistent with its fibrous architecture^54^. Phenomenological approaches employ transversely isotropic models consisting of parallel myofibers embedded in an isotropic matrix^55,56^, whereas more structurally-motivated descriptions use the “fiber-sheet-normal” local coordinate system^57–59^ supported by histological studies^60^. Experimentation with passive myocardium under biaxial testing has demonstrated the pronounced nonlinearity, viscoelasticity, and anisotropy of the tissue^61^, and these features have been consistently reported at the tissue level^55,56,62,63^. More recent studies using full 3D kinematics have demonstrated unique mechanical coupling behaviors not previously observed^14^. These myocardial behaviors are largely results of the limitations in considering myocardium with a continuum approach. Given the structural and functional complexity of the myocardium and the results of the current study, analyses of the heart that assume incompressibility may require a re-evaluation^64^.

The relationship between the action potential and contraction force is complex^65^ and has been the subject of diverse experimental studies^66,67^. Direct coupling of electrophysiology with mechanics using ionic models poses many difficulties, particularly in the specification of the constants governing biophysical phenomena, as well as their initial and boundary conditions^68^. Furthermore, significant stresses develop in the cross-fiber direction as a manifestation of the contractile process itself^16^ consistent with lateral force generation in other striated muscles and cross-bridge models^14,69^, yet these effects remain understudied in the heart. To make our heart-specific model tractable, we utilized measured MAP data to provide a first estimate of spatiotemporally varying contraction kinetics. We used a beta distribution to approximate local cytosolic calcium dynamics whose peak (the time of maximum contraction) was defined as the time of 60% decay of the MAP and whose width was related to the re-polarization time, based on experimental recordings^65,70^. Although this simplification is a limitation of our study, this methodology increases realism compared to mechanical models employing synchronous contraction patterns^71^. A further limitation is that the unloaded heart is not completely stress-free due to the presence of residual stresses^72,73^. Residual stress is an active area of research and its inclusion is planned for subsequent versions of our modeling effort.

In conclusion, this study has demonstrated that utilization of myocardial compressibility can improve organ-level simulation accuracy of the deformation patterns of LV. More importantly, use of the compressible myocardial material model revealed profound effects on the *relative* changes in predicted active contraction forces between the pre- and post-MI states. While not directly verifiable, our findings suggest that use of a compressible material model improves our ability to predict more realistic relative changes in contractile forces in the remaining normal myocardium post-MI. Accurate force generation is important as it is a key determinant of borderzone growth and remodeling in the post-MI heart. The compressible tissue modeling approach presented in this work therefore contributes to a more complete representation of the physiological function of heart in heath and disease.

## Methods

### Experimental protocol

All data collection for the development of the in silico heart model was performed at the Visible Heart Laboratory (VHL, University of Minnesota), in three steps: (i) in vivo, (ii) ex situ, and (iii) ex vivo. Anteroapical MI was in a total of ten adult Dorset sheep (weight around 40–50 kg) by ligation of the left anterior descending (LAD) and D2 coronary arteries following surgical protocols (Fig. 1a). All experiments were carried out in accordance with relevant guidelines and regulations as described in the Methods section. The surgical and experimental protocols were approved by Institutional Animal Care and Use Committee (IACUC) at the University of Pennsylvania (Protocol 13021226).

In the one animal used for construction of our sheep-specific model, LV pressure *P* was measured with a balloon pressure catheter placed through the left carotid and aortic valve. Distance segments were generated using sonomicrometry crystals placed on the LV base, LV apex, and LV anterior and posterior midwall. Measurements were taken at 500 Hz. Approximations of LV volume were computed using a half-elliptical volume *V*, with the LV apex-to-base distance as the long axis *a* and the anterior-to-posterior LV wall distance as the short axis diameter *b*, with $V=\pi ab^2/6$. Heart rate (*HR*) was obtained from the temporal lag in between two QRS complexes measured with standard echocardiography, and time was non-dimensionalized to $t\in [0,1]$. End-diastole was chosen as time $t=0$ and was identified using the lower-right corner of the pressure–volume loop. Nine consecutive heart cycles were chosen to obtain a representative LV PV loop by averaging. Standard analysis of the PV loop allowed the identification of end-diastolic and end-systolic volumes (*EDV* and *ESV*, respectively) and yielded standard cardiac function metrics, such as stroke volume $SV=EDV-ESV$, ejection fraction $EF = SV/EDV$, and cardiac output $CO=SV*HR$. We defined stroke work as the area inside the PV loop and identified maximal changes of pressure $dP/dt_{max}$, which occur early during isovolumic contraction and correlate with cardiac contractility, and minimal (negative) changes of pressure $dP/dt_{min}$, which occur shortly after the aortic valve closes and quantify isovolumic relaxation.

For validation of the biventricular model, echocardiographic images were acquired with corresponding electrocardiogram (ECG) data. We chose representative end-diastole (ED) and end-systole (ES) frames at the maximum volume and at the minimum volume, respectively. Frames were collected at long axis, short axis, and four-chamber long axis views. We measured, averaged, and recorded key features in all frame such as LV height. These measurements were taken in units of pixels within ImageJ software, using irregular area and straight-line selection tools. We normalized each measurement with respect its value in the ED frame. After the heart was excised, it was cleaned and then perfusion fixed in 10% buffered formalin under end-diastolic pressure (around 15 mmHg) with valves coapted. MRI was subsequently performed to obtain the anatomy of heart at end-diastole. Following MRI, the samples were kept at 4 $^\circ$C and then scanned with DTMRI.

### LV free wall volume change and thickness measurements

As described in our previous paper, nine sheep underwent sonocrystal implantation at time of MI surgery to measure compressibility in vivo^22^. The sonocrystal array spanned the entire LV free wall and was composed by 27 sonocrystals placed in the endocardium, epicardium, and mid-wall (Fig. 1a). Sonocrystal $\{x,y,z\}$ coordinates captured at multiple cardiac cycles at 5 horizontal time points: pre- and post-infarct (90 min) and subsequently at t = 2, 4, and 8 weeks after infarction. The positions of the sonocrystals during the cardiac cycle in vivo yielded direct measurements of the changes of the volume of myocardial tissue delimited by the markers (Fig. 1b).The domain consisted of sonocrystals array was discretized and interpolated by spline function using a convective curvilinear coordinate system. The volume changes was defined as $J=\Delta v / \Delta V$, where $\Delta v$ is the reference volume and $\Delta V$ is the local volume in current configuration.

We analysed wall thickness changes in the LV free wall using the sonomicrometry measurements. We used the sonomicrometry study for validation instead of imaging data to avoid imaging artifacts and provide more accurate tracking of wall thickness changes at predetermined physical points. In this study, twelve sonocrystals were implanted on the epicardium and nine sonocrystals were inserted on the endocardium. Based on the locations of sonocrystals on the epicardium, a surface mesh was created each time step. Then, all nine crystals on the endocardium were projected onto the surface mesh and distances were calculated for each time frame, which were treated as wall thickness. Since some sonocrystals (1–3) around the apex were too close to epicardium, large variations appeared and so only six endocardial sonocrystals (4–9) were used to represent wall thickness change. The validation data for wall thickness change was generated by averaging values over 10 ovine heart for groups. The same technique was applied to wall thickness calculation for the in-silico heart models. We tracked a total of 845 nodes on finite element mesh on endocardium over one cardiac cycle and wall thickness changes were averaged based on these values.

### Constitutive modeling of myocardium

We utilized a conventional incompressible transversely isotropic Fung-based hyperelastic model for the passive mechanical properties of myocardium, where the Cauchy stress $\mathbf {T}$ is given by the form

1 $$\begin{aligned} \mathbf {T} = -\frac{\partial W^{vol}}{\partial J} + \frac{1}{J}\tilde{\mathbf {F}}\frac{\partial W^{dev}}{\partial \tilde{\mathbf {F}}}\tilde{\mathbf {F}}^{\mathrm{T}} + \frac{1}{J}\mathbf {F}S^{act}\mathbf {F}^{\mathrm{T}}, \end{aligned}$$

where $\mathbf {F}$ is the deformation gradient, the deviatoric deformation gradient is defined as

2 $$\begin{aligned} \tilde{\mathbf {F}} = J^{-1/3}\mathbf {F}, \end{aligned}$$

the Jacobian of the motion is defined as

3 $$\begin{aligned} J = \det \mathbf {F}, \end{aligned}$$

and the deviatoric Green–Lagrange strain is

4 $$\begin{aligned} \tilde{\mathbf {E}} = \frac{1}{2}(\tilde{\mathbf {F}}^{\mathrm{T}}\tilde{\mathbf {F}}-\mathbf {I}). \end{aligned}$$

The passive mechanics of the myocardium are described by its volumetric and deviatoric responses, given by

5 $$\begin{aligned} W^{vol} = \frac{K}{2}\left( \frac{J^2-1}{2} - \ln J \right) \end{aligned}$$

and

6 $$\begin{aligned} W^{dev} = \frac{c}{2}[\exp (\alpha \tilde{\mathbf {E}} \cdot C\tilde{\mathbf {E}}) - 1] \end{aligned}$$

respectively, where *K* is the bulk modulus (with $K \gg c$ enforcing material incompressibility). The constants *c* and $\alpha$ are passive material parameters, and fourth-order tensor C characterizes the transverse isotropy of the material, specifically resulting in

7 $$\begin{aligned} W^{dev} = \frac{c}{2}[\exp (\alpha Q) - 1], \end{aligned}$$

where

8 $$\begin{aligned} Q = A_1{E_{11}^2} + A_2 \left( {E_{22}^2} + {E_{33}^2} + 2{E_{23}^2} \right) + A_3 \left( {E_{12}^2} + {E_{13}^2} \right) , \end{aligned}$$

with $A_1 = 12.0$, $A_2 = 8.0$, and $A_3 = 26.0$^23^. The passive model parameters *c* and $\alpha$ are calibrated to match the general end-diastolic pressure volume relationship obtained by Klotz et al.^24^.

The active stress responsible for myocardium contraction follows the Hunter–McCulloch–Ter Keurs (HMT) model^25^, with some minor modifications, is given by

9 $$\begin{aligned} S^{act} = T_{\mathrm{Ca}^{2+}}(t)f(x,t)[1+\beta (\lambda - 1)]/\lambda ^{2} \mathbf {m} \otimes \mathbf {m}, \end{aligned}$$

where $\mathbf {m}$ is the fiber direction, $\lambda$ is the local fiber stretch given by

10 $$\begin{aligned} \lambda = \sqrt{\mathbf {m} \cdot \mathbf {F}^\text {T}\mathbf {F} \mathbf {m}}, \end{aligned}$$

and f(x,t) represents monophasic action potentials varying in time and locations which were measured using surface electrodes in vivo. f(x,t) tales the same role as calcium concentration on active contraction in classic HMT model. The other terms in (9) control the correlation of fiber length with active contraction force.

To account for the distinctive type of compressible material behavior (i.e., incompressible to volume changes $J > 1$ but compressible when actively contracting), we developed a specialized material model that (i) utilizes a penalty term allowing for a reduction in volume only and (ii) is modulated by the active contraction. Specifically, we enforced material incompressibility to passive mechanical deformations that involve volumetric changes (i.e., $J = 1$ is enforced whenever $T_{\mathrm{Ca}^{2+}}(t) = 0$); however, we chose a functional form for bulk modulus *K* dependent on the amount of active contraction, i.e.,

11 $$\begin{aligned} K \left( T_{\mathrm{Ca}^{2+}} \right) =K^{inc} - \gamma T_{\mathrm{Ca}^{2+}}, \end{aligned}$$

such that the material becomes compressible during active systole, and reductions in volume occur.

### In silico heart model generation pipeline

The pressure–volume (PV) loop, measured in vivo through catheterization and sonomicrometry from the infarcted heart, was used to fit the modulation of active contraction in all cases to allow direct comparison of results (Fig. 3). The measurement locations were employed to define an interpolating triangulation, and subsequently, the MAP waveform at each individual element, was obtained through triangulation with barycentric coordinates. The active stress responsible for myocardium contraction followed the well-established Hunter–McCulloch–Ter Keurs model along the fiber directions defined locally with DTMRI^25^. A total of 71 measurements (over the cardiac cycle, synchronized with the QRS complex of ECG) were taken in scattered locations throughout the LV and RV epicardium. We used measured electrical data to spatially and temporally modulate the heterogeneity of active contraction. When modeling MI, contraction was shut down in the infarcted area with no mechanical property changes in the myocardium.

### Simulation methods

The finite element mesh were created in Trelis (Csimsoft Inc.) using 10-node tretrahedral elements and it was treated as input file into Abaqus (Dassault Systemes, 2017). The constitutive model of myocardium was written in UMAT which contains passive behavior following Fung’s orthotropic model, active contraction fitted by MAP waveforms and volumetric response in Eq. (5). The kinematic boundary condition of current model is defined as pressure applied on LV and RV cavities. External soft tissue was built above current model and top plane was fixed in up-down movement. Meanwhile, one point on the top plane was fixed in all direction and the other point was restricted by rotation. The FE analysis took advantage of implicit solver in Abaqus/Standard which used backward Euler integration scheme.

### In silico heart model fitting methods

Passive mechanics parameters were calibrated to the collected ED pressure–volume point and were validated based on large animal and human work done by Wang et al.^23^ parameters and Klotz et al.^24^. Starting from the ED ventricular geometry, the FE heart model was unloaded to a stress-free configuration and then inflated to ED pressure. This step was performed iteratively to match the FE model curve with the EDPVR curve. The fitted passive model matched the general EDPVR with a difference of 0.53 mL in end-diastolic volume (Fig. 2), and the fitted constants (namely, $c = 1.522$ kPa) were in agreement with previously published experimental and computational results^23,27–30^.

The global active contraction parameter $T_{\mathrm{Ca}^{2+}}(t)$ was estimated with the fitted passive properties from the EDPVR and a full cardiac cycle of pressure–volume data. The starting point was from the ED point, with an initial value of $T_{\mathrm{Ca}^{2+}}(0) = 0.0$. For each time point on one cardiac cycle, volume change resulting from active contraction compensated for the difference between volume from experimental measurements and volume calculated from passive inflation followed by ventricular pressure.

Specifically, we enforced material incompressibility of passive mechanical deformations (achieved with a much higher volumetric stiffness when compared with the deviatoric stiffness); however, a functional form for bulk modulus was modeled as a function that is dependent on the current and local magnitude of active contraction, such that the material becomes compressible during active systole and reductions in volume can occur. Because we were interested in modeling changes from end-diastole to peak-systole, a linearly decreasing function from the very high bulk modulus of nearly-incompressible passive myocardium with increasing active contraction magnitude was sufficient to obtain the reductions in volume observed from end-diastole to peak-systole in our sonocrystal studies.

### ARRIVE guideline compliance

All in-vivo data that was utilized in this work was followed the ARRIVE guidelines.

## Supplementary Information

Supplementary Information 1.

Supplementary Information 2.
