# Supplementary material for: The impact of myocardial compressibility on organ-level simulations of the normal and infarcted heart
Source: Sci Rep. 2021 Jun 29;11:13466. doi: 10.1038/s41598-021-92810-y (PMC8242073; doi:10.1038/s41598-021-92810-y)
Supplement: Supplementary file 2 — Supplementary Information 2. [file 41598_2021_92810_MOESM2_ESM.pdf]

The Impact of Myocardial Compressibility on Organ-Level Simulations of the Normal and Infarcted Heart

Hao Liu[1,+], Joao S. Soares[2,+], John Walmsley[1], David S. Li[1], Samarth Raut[1], Reza Avazmohammadi[3], Paul Iaizzo[4], Mark Palmer[6], Joseph H. Gorman III[5], Robert C. Gorman[5], and Michael S. Sacks[1,*]

1 James T. Willerson Center for Cardiovascular Modeling and Simulation, The University of Texas at Austin, Austin, TX, USA
2 Engineered Tissue Multiscale Mechanics and Modeling Laboratory, Virginia Commonwealth University, Richmond, VA, USA
3 Computational Cardiovascular Bioengineering Lab, Texas A&M University, College Station, TX, USA
4 Visible Heart Lab, University of Minnesota Twin Cities, Minneapolis, MN, USA
5 Gorman Cardiovascular Research Group, University of Pennsylvania, Philadelphia, PA, USA
6 Corporate Core Technologies, Medtronic, Inc.
* msacks@oden.utexas.edu
+Both authors contributed equally to this work

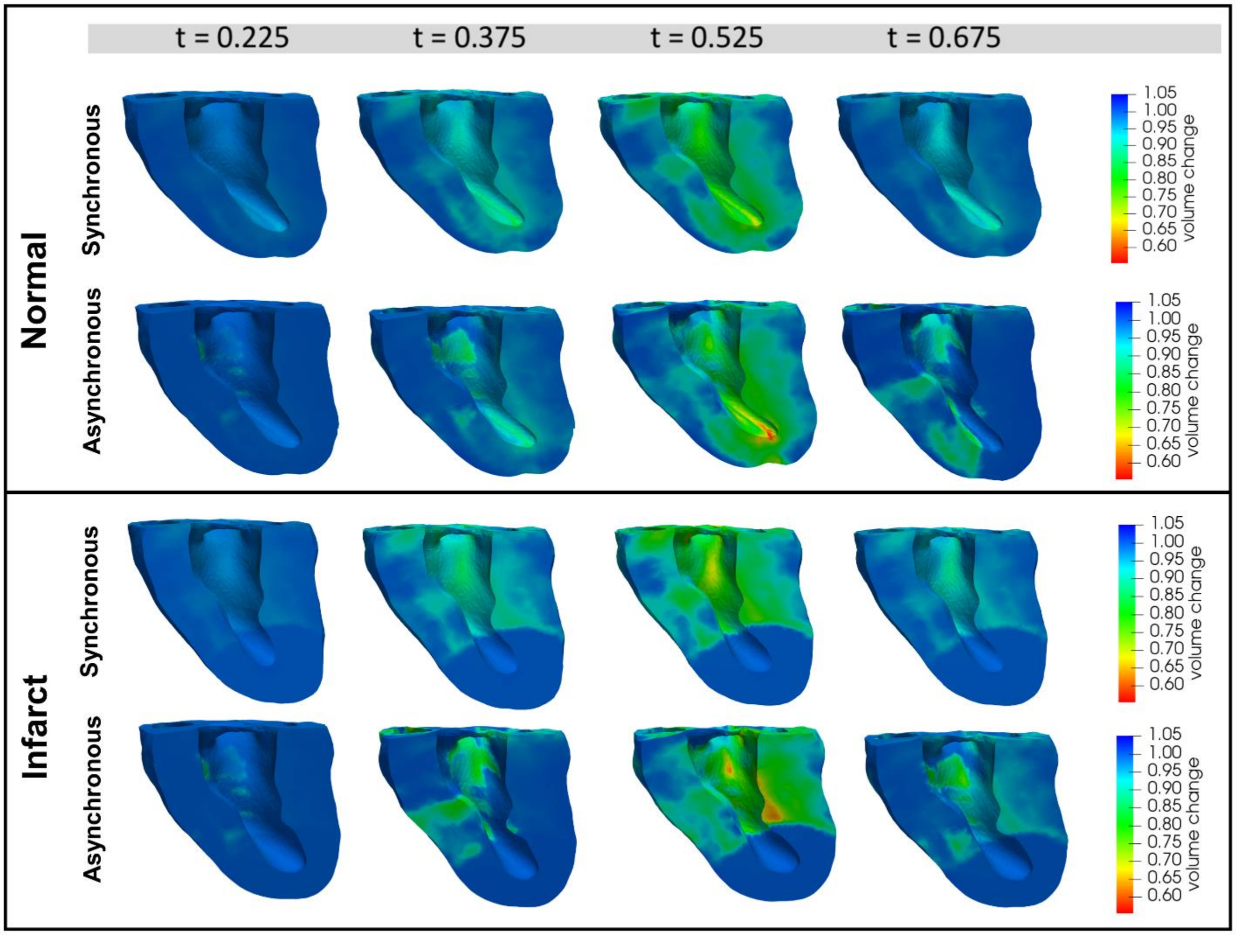

Figure S1. Comparisons of volume reduction in one cardiac cycle between synchronous and asynchronous cases in both normal and infarct in-silico heart models
